# Ulceration of the Vagina Connected with the States of Utero Gestation and Lactation

**Published:** 1850-05

**Authors:** 


*Ulcerations of the Vagina, connected with the states of Utero-Gestation and Lactation.* By DANIEL BRAINARD, M. D., Professor of Surgery in Rush Medical College.

The "nursing sore mouth" is a disease which has only of late attracted the notice of medical writers; yet its pathology and treatment have been investigated with zeal, if not with entire success. It is certainly surprising that such an affection should so long have escaped the notice of observers, if it existed; and equally strange, it may appear, that it should have originated in these latter times. We are inclined to the latter opinion, and suppose that it is on the increase, both as regards its frequency and its severity. These ulcerations, however, are to be regarded only as a local effect of a general cause, which does not by any means confine its influence to the mucous membrane of the mouth, but which also as often produces similar effects upon the vaginal surface, and apparently on that of the small intestines.

The state of the system which gives rise to these ulcerations, is anæmia. Those who have been bled often, or confined to a low diet, or affected with a diarrhœa, or frequently purged, are the persons affected. It is usually attended by a leucophlegmatic state, pallor of all the tissues, costiveness or diarrhœa, and frequent desire to urinate, with smarting pain on urination. In the Western States the diarrhœa usually attacks persons recently arrived from the Eastern States or foreign countries, and is often persistent, and even dangerous. Women in the states of gestation, or nursing, who labor under this affection, are generally attacked with these mucous ulcerations.

The causes of the disease have already been stated to be of a debilitating nature. Lactation, when prolonged, and accompanied by an insufficient nourishment, is by far the most frequent; hence its name, "nursing sore mouth."

The treatment most effectual, verifies this view of the case. A general course of tonics, with nourishing and abundant food, with free exercise in the open air, seldom fail to afford relief. Good beer, ale or porter, with beef and mutton, are the best means to employ. Iron, and Vegetable Bitters, are of some service, particularly the former. As a local application to the ulcerations of the mouth, no remedy deserves to be compared to the fuming Muriatic Acid, applied with a probe, piece of wood, or brush, to the ulcerated surface; it never fails to relieve when the ulcers are white and circumscribed. When there is a diffused redness and denudation, it should be diluted and used as a wash. Mercurials are especially to be avoided.

To illustrate these brief and very imperfect remarks, I will add some cases which may be taken as specimens of the different forms in which it appears.

Case 1. Mrs. A., a young woman of scrofulous habit and delicate constitution, was affected, while pregnant with her first child, with ulcers of the mouth, for which she made use of astringent applications. After using these the mouth was cured; but ulcerations of a very severe kind attacked the genital organs, there being several deep and whitish ulcerated patches about the orifice of the urethra and vagina, which produced great pain and smarting on urination, and pain in the hip, groin, and extending down the thighs. There was considerable constitutional irritation, which soon became severe. Local applications had little effect, and the ulcerations continued until delivery, when they disappeared and the mouth became affected, continuing with varying degrees of intensity during the whole period of lactation. At the second pregnancy and lactation, the disease reappeared in so severe a form as to endanger her life, and render necessary the induction of premature labor, when it again ceased and attacked her mouth.

Case II. Mrs. C., a young woman of delicate constitution, had, during pregnancy and lactation with her first children, ulcers of the mouth. During the pregnancy and lactation with the third child, it recurred, and was treated by the application of strong Muriatic Acid. This immediately cured the ulcers, but similar spots made their appearance about the orifice of the vagina, occasioning great smarting, with pain in the hip and groin of the side most affected. This appearance of ulcers of the mouth at different times, was attended with great relief to the other symptoms; but on their healing, the ulcers of the vagina were again seen, with their attendant effects.

Case III. A woman of about 35 years of age, had been affected for a long time with a pain in the back, hips, &c., for which various remedies had been used without effect. On enquiry I found the symptoms dated from the period of lactation, and were attended with debility. On examination, several minute points were seen about the orifice of the vagina, scarcely perceptible to the eye, but which, when the surface was touched with a solution of Lunar Caustic, turned white, revealing the existence of numerous ulcerated points. The appearance of minute red points upon the mucous surface, of a pale color, I have seen in other cases, and it is well calculated to deceive, unless a solution of Nit. Arg., of the strength of about 20 grs. to the oz., is passed on the surface. That is the form of application preferred for this situation, the Muriatic Acid being too severe. It were easy to add to these cases, others, where the ulceration of the mouth alternated with diarrhœa, indicating a transfer of the ulceration from the intestinal mucous membrane to that of the mouth, and the reverse. But we are content with simply inviting the attention of the profession to certain relations of these affections, in order that the same connexion may be observed if it occurs elsewhere.—*N. W. Journal.*

